# Genetic correlation for alcohol consumption between Europeans and East Asians

**DOI:** 10.1186/s12864-023-09766-8

**Published:** 2023-10-30

**Authors:** Xuan Liu, Yongang Li

**Affiliations:** Department of Neurology, The First People’s Hospital of Wenling, Taizhou, China

**Keywords:** Alcohol consumption, Genetic architecture, Genetic correlation, Multi-ancestry meta-analysis

## Abstract

Genome-wide association studies (GWAS) have identified many genetic variants associated with alcohol consumption in Europeans and East Asians, as well as other populations. However, the genetic homogeneity and heterogeneity between these populations have not been thoroughly investigated, despite evidence of varying effect sizes of variants between ethnicities and the presence of population-specific strong signals of selection on loci associated with alcohol consumption. In order to better understand the relationship between Europeans and East Asians in the genetic architecture of alcohol consumption, we compared their heritability and evaluated their genetic correlation using GWAS results from UK Biobank (UKB) and Biobank Japan (BBJ). We found that these two populations have low genetic correlation due to the large difference on chromosome 12. After excluding this chromosome, the genetic correlation was moderately high (${r}_{g}$ = 0.544, p = 1.12e-4) and 44.31% of the genome-wide causal variants were inferred to be shared between Europeans and East Asians. Given those observations, we conducted a meta-analysis on UKB and BBJ and identified new signals, including the CADM2 gene on chromosome 3, which has been associated with various behavioral and metabolic traits. Overall, our findings suggest that the genetic architecture of alcohol consumption is largely shared between Europeans and East Asians, but there are exceptions such as the enrichment of heritability on chromosome 12 in East Asians.

**Supplementary Information:**

The online version contains supplementary material available at 10.1186/s12864-023-09766-8.

## Introduction

Rapid social progress is improving the overall quality of life, with a significant shift in dietary habits. Alcohol, which has traditionally been an important part of human diet, has become a major public health concern in recent years as the relationship between drinking and health issues are better understood. Studies have shown that alcohol consumption is linked to many diseases, including alcohol dependence, cardiovascular and cerebrovascular diseases, digestive system diseases, and other non-communicable diseases and mental diseases. These conditions can seriously impact physical health, quality of life, and even lead to a higher mortality and disability rate than smoking or hypertension [1–4].The International Agency for Research on Cancer (IARC) has classified ethanol and its related metabolites as group 1 carcinogens [5].

Previous genetic association studies have identified genetic loci associated with alcohol consumption and related health issues. For example, Hnrnph1, a novel regulator of alcohol reward driving alcohol consumption and high-dose alcohol aversion, is potentially relevant to the neurobiology of alcohol abuse and alcoholism [6]. Studies have also shown that genetic variations can alter the effect of alcohol intake on the aggressiveness of a range of tumors, such as prostate cancer, colon cancer [7]. For example, SNPs on the CAMK2D, ROBO1, and PRKCA genes, may increase an individual’s vulnerability to excessive alcohol intake and the development of aggressive prostate cancer [8]. And the GA and AA genotypes on rs671 were associated with a lower risk of gout in Asians [9]. These results suggest that genetic factors play an important role in shaping dietary lifestyle as well as overall health.

Although genetic studies on alcohol consumption have largely focused on European populations, studies have also been conducted on individuals of other ancestries, including Asian and African [10, 11]. Large consortiums incorporating individuals from global populations boost the findings of associated genetic loci. The most recent study included a sample size of 3.4 million individuals and identified hundreds of loci associated with alcohol consumption [12].

On the other hand, studies have showed evidence that some of the genetic variants associated with alcohol consumption have different effects between populations. For example, strong associations have been identified at ALHD2 in multiple Asian populations [10, 13, 14], while there is a lack of signals in other populations. Another example is ADH1B. Signals of association at this locus have been observed in both Europeans [13] and East Asians [14], but a meta-analysis across ethnicities shows that its effect on alcohol consumption varies across ethnicities [12]. In addition to the differences in effect size between ethnicities, studies on selection signature have found that the ADH1B gene has undergone positive selection in East Asian populations [15, 16], which differentiated East Asians from other populations in allele frequencies. Although a selection signal has also been identified at this locus in Europeans [17], the signal is likely due to uncorrected population structure [18].

Despite evidence that genetics play a role in alcohol consumption and that its effect varies by ethnicity[12, 13], there has been limited effort to investigate the genetic homogeneity and heterogeneity of alcohol consumption between populations. To fill this gap, we conducted a comprehensively study on the genetic similarities and differences in alcohol consumption between populations, using Europeans and East Asians as representative populations, since they have the largest available GWAS data, that is UK Biobank (UKB) and Biobank Japan (BBJ). We first investigated the impact of chromosome 12, where heritability was largely enriched in East Asians, on the estimation of genetic correlation between the two populations. We also assessed the sharing of causal variants between these two populations and conducted a meta-analysis, which enhanced statistical power and unveiled novel signals.

## Materials and methods

### GWAS panels

We obtained publicly available GWAS summary statistics from two populations: UK Biobank (UKB) and Biobank Japan (BBJ). UKB is a GWAS based on ~ 360,000 individuals of white British ancestry in the UK Biobank. These individuals were genotyped with either the Affymetrix UK BiLEVE Axiom Array or the Affymetrix UK Bio-bank Axiom Array and imputed to whole-genome sequencing data for ~ 13.8 M variants. Association testing was done on drinker status with ~ 11,000 cases (Never drinker) and ~ 350,000 controls (Ever drinker) correcting for age, age^2^, sex, age * sex, age^2^ * sex, and 20 PCs. BBJ is a GWAS based on ~ 165,000 individuals of Japanese ancestry from Biobank Japan. These individuals were genotypes on either the Illumina HumanOmniExpress Exome BeadChip or a combination of the Illumina HumanOmniExpress and HumanExome BeadChips, and phased and imputed with a reference panel constructed from 275 unrelated EAS haplotypes from 1000 Genomes. After adjusting for age, age^2^, sex, and the status of the 45 target diseases registered in BBJ, a GWAS was conducted on drinker status with ~ 83,000 controls and ~ 81,000 cases, with a linear mixed model to control for cryptic relatedness and population structure. SNP positions are according to GRCh37 (hg19).

### Heritability

We employed LD Score Regression (LDSC) [19, 20]to estimate the SNP heritability of alcohol consumption for each population. In this analysis we used only HapMap3 SNPs. We obtained pre-computed LD Scores from the LDSC website, which had been generated using 1000 Genomes data from Europeans and East Asians. We used LD Scores derived from Europeans for UKB and LD Scores derived from East Asians for BBJ. SNP heritability was reported on the scale of liability with a population prevalence of 89.75% and 47.02% for UKB and BBJ. The prevalence rates were calculated as the average across sexes from previous studies [21, 22]. Considering the strong signal on chromosome 12 in BBJ, we calculated partitioned heritability by partitioning variants by chromosomes to determine whether the SNP heritability were enriched on certain chromosomes. In this analysis of heritability partitioned by chromosomes, we computed LD scores using Europeans and East Asians from 1000 Genomes dataset as reference populations for UKB and BBJ, respectively.

### Genetic correlation between populations

The genetic correlation of drinker status between European and East Asians was computed using Popcorn [23]. It estimates the genetic correlation of the same phenotype but in two different populations using genome-wide summary statistics. It reports the genetic-effect correlation across populations, which is the correlation coefficient of per-allele SNP effect sizes, and the genetic-impact correlation, which is the correlation of effect sizes after normalizing on allele frequency. In this study, we only reported genetic-effect correlation. We used precomputed scores for EUR and EAS from the Popcorn website, which used 1000 Genome data from Europeans and East Asians as reference. These scores roughly correspond to the similarity in LD at each SNP across the populations. For this analysis, we used the same prevalence rates as in LDSC. We calculated genome-wide genetic correlation both with and without chromosome 12.

### Sharing of casual variants between populations

We used the tool PESCA [24] to determine the extent to which latent causal variants for alcohol consumption are population-shared or population-specific between Europeans and East Asians. We first used PESCA on UKB and BBJ jointly to infer the genome-wide proportion of causal variants that are population-specific or population-shared. For computational efficiency, PESCA requires first defining linkage disequilibrium (LD) blocks that are approximately independent in both populations and assumes that a SNP in a given block is independent from all SNPs in all other blocks. We used the 1,368 approximately LD-independent blocks across East Asians and Europeans previously defined by Shi et al. [24],and computed the LD matrix of each block using 1000 Genomes data from Europeans and East Asians. We narrowed our analysis to ~ 533 K SNPs that have summary association statistics available in both UKB and BBJ, and that also exist in the reference panel provided by PESCA, which was created by filtering for SNPs with minor allele frequency greater than 0.5 and linkage disequilibrium (${r}^{2}$) less than 0.95 in both Europeans and East Asians. In this step, we used the heritability estimated above without chromosome 12 (0.1259 and 0.0471 in UKB and BBJ, respectively). Using the estimated genome-wide proportions of population-specific and shared causal variants as prior probabilities in an empirical Bayes framework, we then used PESCA with default parameters to estimate the posterior probability of each SNP to be causal in a single population (population-specific) or both populations (shared). We inferred the posterior expected number of population-specific/shared causal SNPs in each LD block by summing all SNPs in the block the per-SNP posterior probabilities of being causal in a single or both populations.

### Multi-ancestry meta-analysis

We conducted a multi-ancestry meta-analysis of UKB and BBJ using the tool MAMA [25]. Compared to other cross-ancestry meta-analysis methods, MAMA accounts for differences in conditional effect, allele frequencies, and LD between GWAS populations. This allows for the identification of novel loci that might not have been discovered when analyzing each set of GWAS summary statistics separately. Before running the meta-analysis, we calculated within- and cross-ancestry LD scores for UKB and BBJ using the script from MAMA package and reference populations EUR and EAS from 1000 Genomes. The window size for LD scores calculations was 1 cM, using the genetic map inferred from 1,000 Genomes Phase 3 haplotypes. Next, we ran MAMA to conduct a multi-ancestry meta-analysis. MAMA estimates the variance-covariance matrices of true genetic signal and estimation error at each SNP across populations by using a variation of LD score regression which includes three terms: LD score, confounding bias, and sampling variation, which should be equal to the squared standard error.

To confirm the signals found in MAMA, we did a secondary meta-analysis using METAL [26]. To combine the evidence for association from UKB and BBJ, we used the approach implemented in METAL that weights the effect size estimates by their standard errors.

## Results

### Heritability and genetic correlation

The heritability of drinker status (Never versus Ever drinker) was assessed in two populations, those of European descent in the UKB and those of East Asians descent in the BBJ. The heritability in the UKB was 0.1279 (se = 0.0136, p = 2.62e-21), which is higher than the estimate of 0.0846 provided at the UKB website which assumed a proxy population prevalence equal to the prevalence rate in the GWAS dataset. The heritability in the BBJ was 0.2355 (se = 0.1867, p = 0.1). It’s bigger than the estimate of 0.1237 in the original study [14], but this difference was not statistically significant due to the large estimation error. Next, we assessed whether heritability was enriched on particular chromosomes. Partitioning heritability by chromosome revealed that chromosome 12 had an unusually high contribution to heritability in the BBJ (14.21 times more than expected based on its number of SNPs). Although the enrichment was not statistically significant (p = 0.23) due to a large standard error of 11.25, the large contribution to heritability could bias genome-wide correlation between Europeans and East Asians, as well as other genome-wide analyses. In contrast, we didn’t observe any large enrichment of heritability in the UKB, which has enrichment ranging from 0.43 to 1.55 across chromosomes. Specifically, the enrichment of heritability on chromosome 12 in the UKB was 0.7.

We then used the Popcorn method to measure genetic correlation between UKB and BBJ. The genome-wide genetic correlation of effect sizes was low (${r}_{g}$ = 0.183, p = 0.025) when including chromosome 12. After excluding chromosome 12, the genetic correlation increased to ${r}_{g}$ = 0.544 (p = 1.12e-4). This suggests that the effect sizes of common variants are highly correlated between UKB and BBJ, except for chromosome 12. This also indicates that the impact from a single chromosome or genetic locus could be significant enough to influence the overall genome-wide coefficients like the genetic correlation between populations, which in turn could lead to inaccuracies and biases in subsequent genome-wide analyses, such as a meta-analysis of GWAS. Given the unusually large heritability in BBJ and the low genetic correlation between UKB and BBJ on chromosome 12, this chromosome was excluded from further analyses to avoid its potential bias effect on other chromosomes.

### Population-specific and shared causal variants

**Fig. 1:**
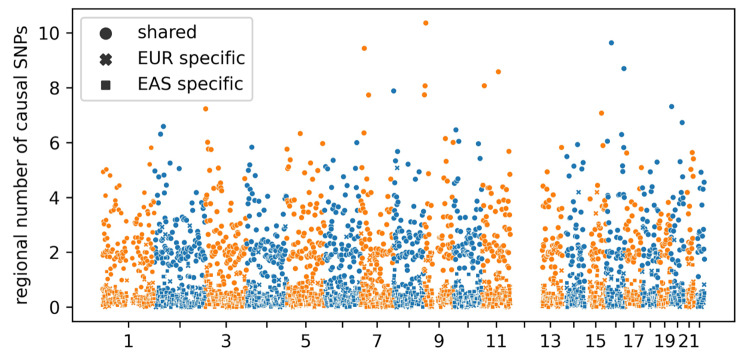
Distributions of the number of population-specific/shared causal SNPs across 1,368 approximately LD-independent blocks along the genome. Each marker represents an independent LD block. For each block, the posterior expected number of SNPs in a given causal configuration is estimated by summing, across all SNPs in the block, the per-SNP posterior probabilities of having that causal configuration

Through the PESCA analysis, we found 0.30% of all common SNPs were inferred to have nonzero effects in both EUR and EAS; 0.28% and 0.14% were inferred to have population-specific nonzero effects in EUR and EAS, respectively. In other words, approximately 41.9% of SNPs inferred to be causal were shared between EUR and EAS. We inferred the posterior expected number of population-specific/shared causal SNPs in each of the 1,368 independent LD blocks by summing all SNPs in the block the per-SNP posterior probabilities of being causal in a single or both populations. High-posterior SNPs for both the population-specific and shared causal configurations were spread nearly uniformly across the genome (Fig. 1), with a mean of 0.37 (SD = 0.29) EAS-specific, 0.37 (SD = 0.44) EUR-specific, and 2.53 (SD = 1.33) shared high-posterior SNPs per region. When examining LD blocks with significant association signals, we found that in the unique LD block (located between 99.4 Mb and 100.7 Mb on chromosome 4) that had significant signals in both UKB and BBJ, there were 4.04 SNPs with high posterior probabilities that were shared between UKB and BBJ, which was higher than the genome-wide average of 2.53. This implies that the associated signals found in UKB and BBJ partially stem from the same causal variants. Aggregating posterior probabilities by chromosome, we find that the posterior expected number of EAS-specific, EUR-specific, and shared causal SNPs per chromosome are highly correlated with chromosome length with ${r}^{2}$ of 0.958 (p = 9.29e-12), 0.947 (p = 7.87e-11), and 0.977 (p = 3.85e-14), respectively.

### Meta-analysis on GWAS summary statistics using MAMA

**Fig. 2:**
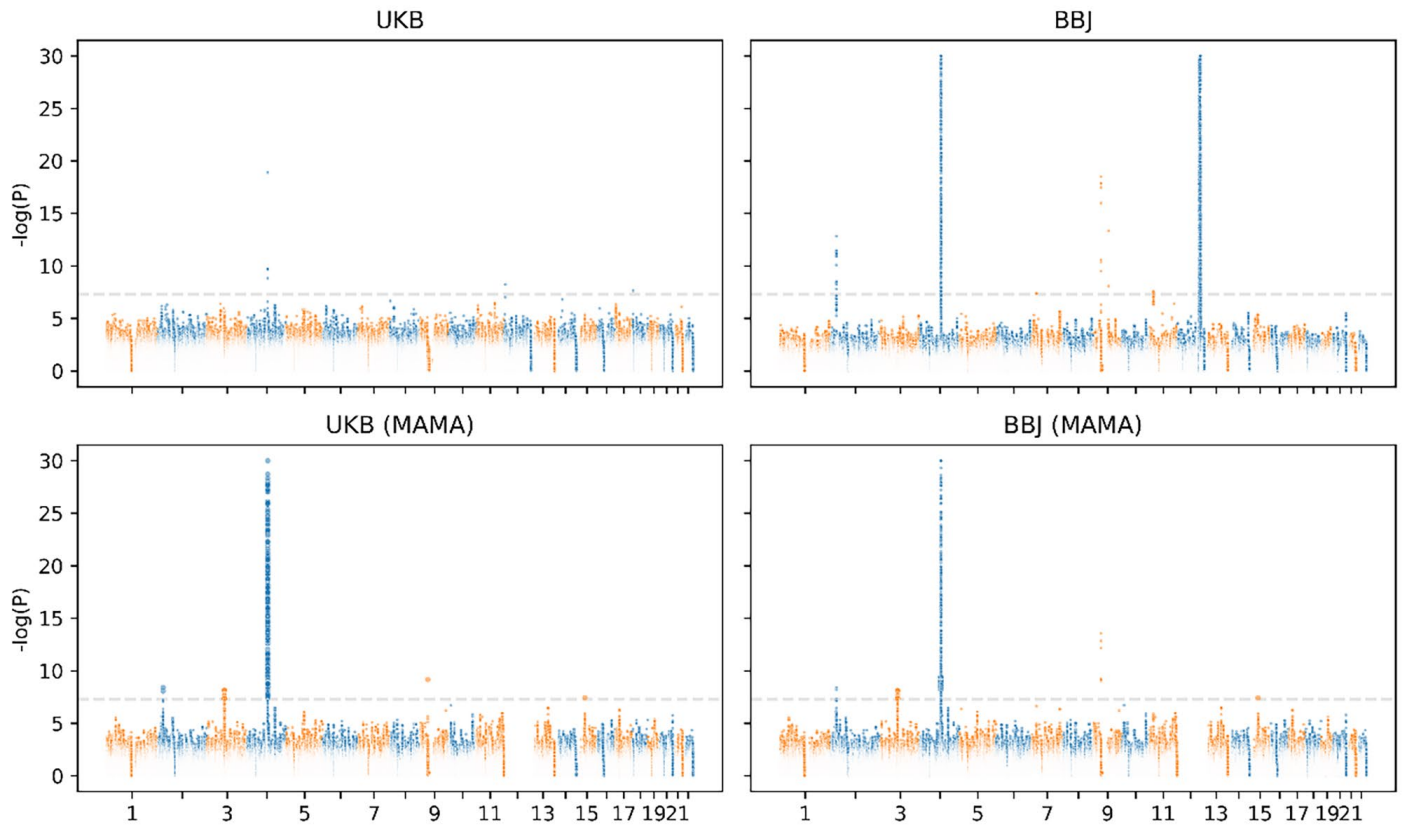
Manhattan plot for genome-wide association signals in UKB and BBJ. The upper panels show results from the initial GWAS; the lower panels show results from MAMA. The large circles in lower panels indicate SNPs that were not significant initially but became significant in MAMA. P values were capped at -log(p) = 30

**Fig. 3:**
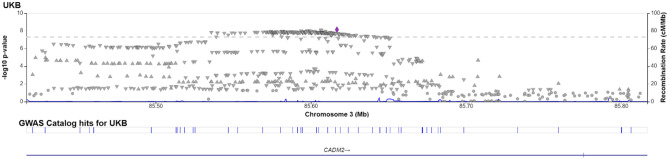
LocusZoom plot for SNP rs1597315 in UKB from MAMA. The purple dot indicates the lead SNP rs1597315 in the region

**Fig. 4:**
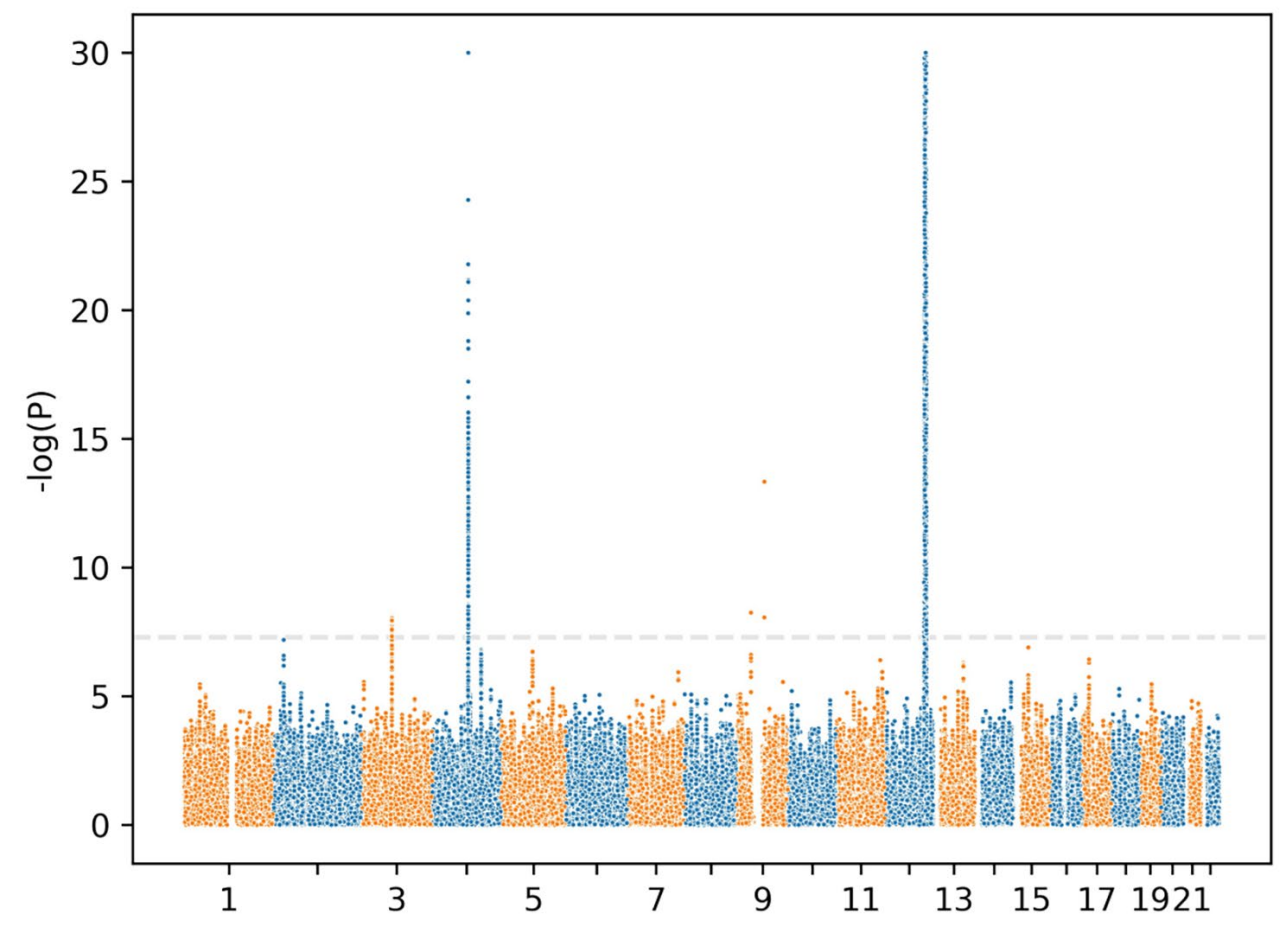
Manhattan plot for genome-wide association signals in a meta-analysis using METAL. P values were capped at -log(p) = 30

Given that most causal variants were shared between Europeans and East Asians, we conducted a meta-analysis of UKB and BBJ using MAMA. The analysis successfully identified new signals that were originally missing in one of the populations or both (Fig. 2). One promising signal was found at a genetic locus on chromosome 3 in both UKB and BBJ, where no signals were originally detected in either UKB or BBJ. The lead SNP rs1597315 has p = 7.87e-9 in BBJ and p = 7.25e-9 in UKB (Table 1). This locus overlapped with the CADM2 gene (Fig. 3 and Figure S1). The CADM2 was used to be reported as a tumor suppressor and is usually downregulated in several cancers [27]. Recent studies show that reducing CADM2 expression can reverse several traits associated with the metabolic syndrome including obesity, insulin resistance, and impaired glucose homeostasis [28]. We also identified another new signal on chromosome 15 that was originally missing in both UKB and BBJ. The lead SNP was rs11634582 with p = 3.76e-8 in both UKB and BBJ (Table 1). However, no genes were found in the locus. Furthermore, MAMA also observed new associated loci in UKB that were originally significant in BBJ only. These loci were located on chromosome 2 in the GCKR gene, on chromosome 4 in the ADH gene cluster containing the well-known ADH1B gene, and on chromosome 9 in the ALDH1B1 gene. We find that the signal on chromosome 3 was replicated in the meta-analysis using METAL with the lead SNP rs2875908 (p = 7.48e-9) at 85,597,693 bp (Fig. 4). Although the signal on chromosome 15 was not replicated, the lead SNP rs11634582 was close to the genome-wide significance threshold with p = 1.27e-7.

**Table 1:** Candidate loci found in MAMA

SNP	Position (hg19)	Nearby gene	Alleles (Ref/Alt)	BETA (MAMA BBJ)	BETA (MAMA UKB)	SE. (MAMA BBJ)	SE (MAMA UKB)	P (MAMA BBJ)	P (MAMA UKB)	P(METAL)
rs1597315	3:85616809	CADM2	C/T	-0.0080	-0.0018	0.0014	0.0003	7.87e-9	7.25e-9	1.38e-8
rs11634582	15:41896257	MGA	C/T	0.0181	0.0022	0.0033	0.0004	3.76e-8	3.76e-8	1.27e-7

## Discussion

Recently GWAS using common variants have reported signals of association between many genes and alcohol-related traits, including those involved in alcohol metabolism, such as ADH1B, ADH1C, and ADH4 [29–31]. However, the correlation of genetic factors for alcohol consumption or other alcohol-related traits between populations has not been extensively studied. To investigate the similarity and difference in alcohol consumption between Europeans and East Asians, we analyzed GWAS data from UKB and BBJ.

We observe a predominantly shared genetic architecture for alcohol consumption between Europeans and East Asians, except for chromosome 12, where East Asians show significant enrichment of genetic effects compared to Europeans. This discrepancy aligns with evidence of strong natural selection at the ALDH2 locus on chromosome 12 in Japanese populations [16]. These findings underscore how a population’s demographic history can influence its genetic architecture, emphasizing the importance of inclusive genetic studies to attain a comprehensive understanding of disease mechanisms and promote health equality. On the other hand, the significant impact of chromosome 12 on the overall genome-wide coefficients (heritability and genetic correlation between populations) suggests the necessity for a more adaptable model for the distribution of effect sizes across the genome, rather than a basic distribution such as the Normal distribution. Adopting an appropriate distribution of effect sizes could improve the estimation of effects of individual variants both in association studies, including GWAS and meta-analysis, and the construction of polygenic scores, as shown by Spence et al. [32].

Given the moderately high genetic correlation and the large proportion of causal variants shared between populations, we performed a GWAS meta-analysis on BBJ and UKB. By combining GWAS signals from multiple populations, MAMA successfully identified new signals of association with alcohol consumption that were previously missed in initial GWAS. One such example is the CADM2 gene located on chromosome 3. The role of CADM2 is increasingly being recognized in the behavioral and metabolic traits. Recent studies have found that the CADM2 gene is significantly associated with a wide range of traits, including cognitive, risk taking, and dietary traits [33–35]. CADM2 is strongly co-expressed with several genes involved in GABA and other glutamate neural signaling pathways, such as GABA receptors α1 and β2 (GABRA1, GABRB2) and glutamate receptor metabolism 5 (GRM5). In addition, it was also involved in biological processes by the co-expression with many members of the voltage-gated potassium channel group, including KCNJ9, KCNJ10, KCNB2, and KCNC, as well as the OPCML(opioid binding protein/cell adhesion molecule-like) gene and EPHA5 (EPH receptor A5) [34, 36]. A recent study shows that CADM2 is associated with the alcohol consumption at the gene level [37]. In addition to the CADM2 gene, we also discovered new signals at the GCKR, ADH gene cluster, and ALDH1B1 genes in the UKB that were originally only significant in the BBJ [14]. These findings suggest that most GWAS signals are shared between UKB and BBJ. However, in order to fully uncover these signals in the UKB, larger sample sizes will be required.

Since the majority of genetic studies are conducted in European populations, it is imperative to conduct a more systematic and comprehensive assessment of genetic homozygosity and heterozygosity across different populations. This is essential for enhancing genetic findings and understanding of the transferability of polygenic risk score in underrepresented populations [38]. Our findings suggest that a small number of population-specific genetic signals could skew the genetic correlation between populations and potentially affect the accuracy of genetic studies across ethnicities.

### Electronic supplementary material

Below is the link to the electronic supplementary material.

Supplementary Material 1

## Data Availability

The data in this study are all publicly available, any interested party can access the data through a similar procedure. The URLs for data presented herein are as follows: UK Biobank summary statistics, https://broad-ukb-sumstats-us-east-1.s3.amazonaws.com/round2/additive-tsvs/20117_0.gwas.imputed_v3.both_sexes.tsv.bgz. Biobank Japan summary statistics, https://humandbs.biosciencedbc.jp/files/hum0014/hum0014.v19.drink.v1.zip. LD score regression, https://github.com/bulik/ldsc. PESCA, https://github.com/huwenboshi/pesca. Popcorn, https://github.com/brielin/Popcorn. MAMA, https://github.com/JonJala/mama. Genetic map, https://mathgen.stats.ox.ac.uk/impute/1000GP%20Phase%203%20haplotypes%206%20October%202014.html.

## Abbreviations

GWAS: Genome-wide association study
UKB: UK Biobank
BBJ: Biobank Japan
CADM2: Cell adhesion molecule 2
LD: Linkage disequilibrium
LDSC: Linkage disequilibrium Score Regression
SNP: Single nucleotide polymorphism
MAMA: Multi-ancestry meta-analysis
EAS: East Asian
EUR: European
IARC: International Agency for Research on Cancer
PESCA: Proportion of population-specific and shared causal variants
