# Supplementary material for: Genetic correlation for alcohol consumption between Europeans and East Asians
Source: BMC Genomics. 2023 Oct 30;24:652. doi: 10.1186/s12864-023-09766-8 (PMC10614326; doi:10.1186/s12864-023-09766-8)
Supplement: Supplementary file 1 — Supplementary Material 1 [file 12864_2023_9766_MOESM1_ESM.docx]

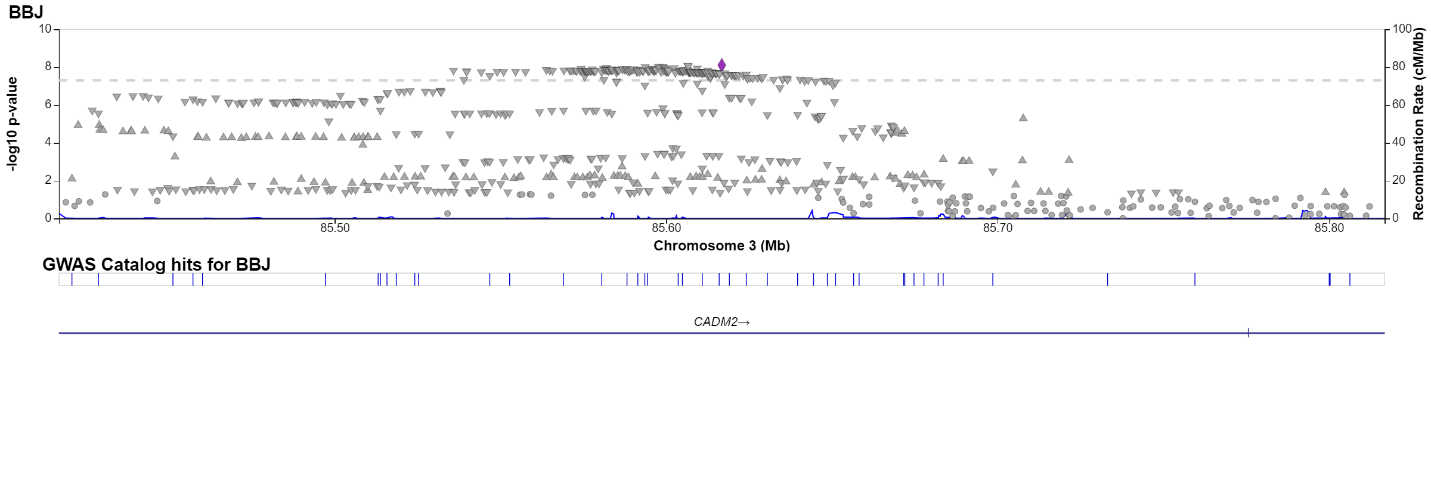


Figure S1. LocusZoom plot for SNP rs1597315 in BBJ from MAMA. The purple dot indicates the lead SNP rs1597315 in the region.
